# Isolation, characterization, and assessment of lactic acid bacteria toward their selection as poultry probiotics

**DOI:** 10.1186/s12866-019-1626-0

**Published:** 2019-11-12

**Authors:** Rine Christopher Reuben, Pravas Chandra Roy, Shovon Lal Sarkar, Rubayet-Ul Alam, Iqbal Kabir Jahid

**Affiliations:** 1 Department of Microbiology, Faculty of Biological Sciences and Technology, Jashore University of Science and Technology, Jashore, 7408 Bangladesh; 2 Department of Science Laboratory Technology, Nasarawa State Polytechnic, P.M.B 109, Lafia, Nigeria

**Keywords:** Probiotics, Lactic acid bacteria, Antagonistic activity, Poultry

## Abstract

**Background:**

Probiotics are live microorganisms that, when administered in adequate amounts, confer a health benefit on the host, are now accepted as suitable alternatives to antibiotics in the control of animal infections and improving animal production. Lactic acid bacteria (LAB) with remarkable functional properties have been evaluated in different studies as possible probiotic candidates. The purpose of this study was to isolate, characterize and assess the potentials of LAB from poultry gastrointestinal tract as potential poultry probiotics.

**Results:**

Potential LAB probiotics were isolated from broilers, characterized and evaluated for probiotic properties including antagonistic activity (against *Escherichia coli*, *E. coli* O157: H7, *Enterococcus faecalis*, *Salmonella* Typhimurium, *S.* Enteritidis and *Listeria monocytogenes*), survivability in simulated gastric juice, tolerance to phenol and bile salts, adhesion to ileum epithelial cells, auto and co-aggregation, hydrophobicity, α–glucosidase inhibitory activity, and antibiotic susceptibility tests. Most promising LAB strains with excellent probiotic potentials were identified by API 50 CHL and 16S rRNA sequencing as *Lactobacillus reuteri* I2, *Pediococcus acidilactici* I5, *P. acidilactici* I8, *P. acidilactici* c3, *P. pentosaceus* I13, and *Enterococcus faecium* c14. They inhibited all the pathogens tested with zones of inhibition ranging from 12.5 ± 0.71 to 20 ± 0 mm, and competitively excluded (*P* < 0.05) the pathogens examined while adhering to ileum epithelial cells with viable counts of 3.0 to 6.0 Log CFU/ml. The selected LAB strains also showed significant (*P* < 0.005) auto and co-aggregation abilities with α-glucosidase inhibitory activity ranging from 12.5 to 92.0%. The antibiotic susceptibility test showed 100.00% resistance of the LAB strains to oxacillin, with multiple antibiotic resistance indices above 0.5.

**Conclusion:**

The selected LAB strains are ideal probiotic candidates which can be applied in the field for the improvement of poultry performance and control of pathogens in poultry, hence curtailing further transmission to humans.

## Background

Over the last decades, increased attention has been given by researchers on the health benefits of microbial species inhabiting animals including humans. The reason is that intestinal microbiota is believed to be the largest bacterial reservoir in animals [1]. These beneficial microbial strains collectively referred to as probiotics are known to be “live microorganisms that, when administered in adequate amounts, confer a health benefit on the host” [2].

Therefore, the application of native strains of lactic acid bacteria (LAB) as animals’ probiotics could provide most suitable substitute for the control and prevention of animals’ diseases [3]. LAB including species of *Enterococcus, Lactobacillus, Pediococcus, Streptococcus, Lactococcus, Vagococcus, Leuconostoc, Oenococcus, Weissella, Carnobacterium* and *Tetragenococcus* are natural microflora of both humans and animals GIT [4, 5]. They have been reported to possess a broad spectrum of beneficial and health promoting properties which dramatically influences the host microbial intestinal balance and general performance [6, 7]. Although the effectiveness of LAB strains used as probiotics are species and/or strains dependent, they must nevertheless meet all the necessary criteria needed for acceptability as probiotics as earlier described [8].

Feed supplementation with different types of antibiotics used as growth promoters and therapeutic agents for enhancing poultry’s performance is a widespread practice in Bangladesh and most countries. Consequently, with the resultant public health concerns including the emergence and spread of antibiotic-resistant strains of bacteria, and the presence of low concentrations of antibiotics in broiler meat, antibiotic supplementation has raised serious concerns lately [9]. Furthermore, regulatory pressures over the years have limited the application of antibiotics in poultry and livestock production mostly in developed countries [10].

The continuous growing demand for organic farming and the reduction of antimicrobial usage in food-producing animals obviously necessitate the intense search for novel alternatives, including new probiotic strains with more effective properties most especially in curtailing diseases and improving production. Presently, different commercial probiotic products marketed globally are available for poultry. Nevertheless, some of them may not be highly potent due to insufficient examination of the specific beneficial properties of the probiotic strains formulated in the product [11]. Moreover, most manufacturers lack the patience to conduct an in-depth study of each strain to ascertain its full probiotic potentials before commercializing as most industries are after profits maximization with minimal expense.

Currently, poultry production is increasing in developing countries including Bangladesh [12]. With the pressure placed on antibiotics in poultry production and its consequent public health dangers, there is an increasing demand for poultry probiotics in Bangladesh. Unfortunately, available commercial probiotics marketed are readily imported, with huge fortunes expended. As such, this research will unravel novel LAB strains with best probiotic properties which will be used in poultry and subsequently formulated for large-scale industrial production and commercialized for poultry farmers in and outside Bangladesh. Therefore, this study was designed to isolate, characterize, and assess LAB strains with optimal probiotic properties from broilers GIT for supplementation as poultry probiotics.

## Results

**Fig. 1:**
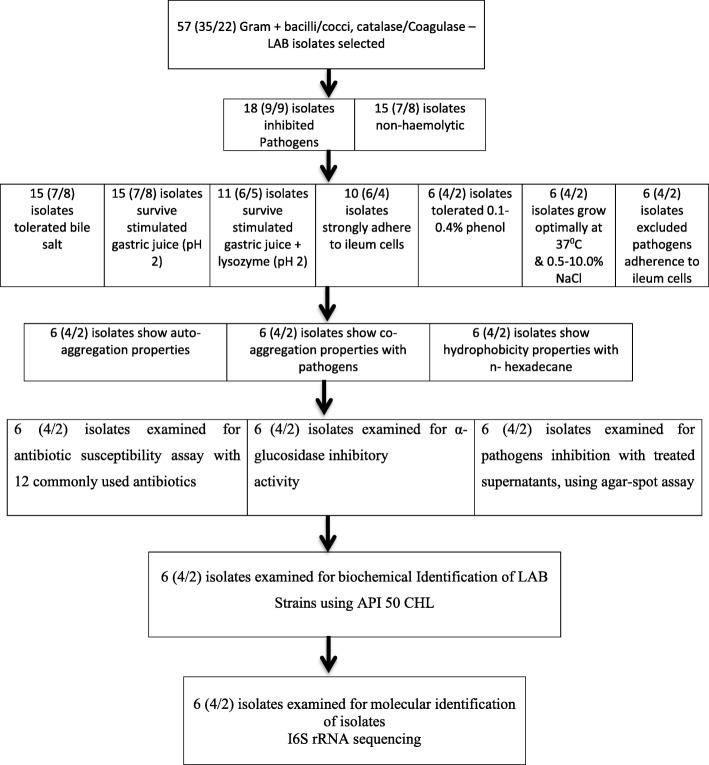
Strain selection flow chart by phenotypic and genotypic methods. Strains without desired probiotics properties were excluded from subsequent examination. LAB: lactic acid bacteria

The criteria for identifying and selecting the most promising LAB probiotic strains to be used as poultry probiotics are shown in Fig. 1.

### Isolation of LAB

In total, 57 LAB strains were isolated from the GIT of apparently healthy broiler chickens (with 35 and 22 from the intestine and crop respectively) based on their typical morphological appearance (small pinpointed and creamy white colonies), Gram-positive, catalase and coagulase-negative and non-motile, coccus and rod-shaped characteristics [13].

### Antagonistic activity

#### Agar well diffusion method

Antagonistic activity against 6 indicator strains (pathogens) including *Escherichia coli* ATCC 10536, *E. coli* O157: H7 ATCC 43894, *Enterococus faecalis* ATCC 51299, *Salmonella* Typhimurium ATCC 14028, *S.* Enteritidis ATCC 13098 and *Listeria monocytogenes* ATCC 19113 was tested with the 57 LAB isolates. Using the agar well diffusion assay, 18 LAB isolates (9 each from both intestine and crop) displayed inhibition activities against all the pathogens tested at different degrees (Table 1). Wider zones of inhibition were exhibited by LAB isolates against *E. coli* ranging between 17 ± 0 to 20.0 ± 0 mm while the least zones of inhibition ranging from 12.5 ± 0.71 to 17 ± 0 mm as revealed from this study were recorded against *S.* Enteritidis. Furthermore, LAB isolates from the intestines showed wider inhibition zones against indicator pathogens examined.

**Table 1:** Antagonistic activity of potential lactic acid bacteria (LAB) probiotic strains from broiler GIT against pathogenic bacteria by agar well diffusion technique

Zone of inhibition (mm)						
Strain	*Escherichia coli*	*Escherichia coli* O157: H7	*Enterococcus faecalis*	*Salmonella* Typhimurium	*Salmonella* Enteritidis	*Listeria monocytogenes*
I1	19.5 ± 0.71	17 ± 0	16 ± 1.41	16 ± 0	15.5 ± 0.71	17 ± 1.41
I2	19 ± 1.41	17.5 ± 0.71	16.5 ± 0.71	15.5 ± 0.71	14.5 ± 0.71	16 ± 1.41
I4	19 ± 0	14.5 ± 0.71	16.5 ± 0.71	14.5 ± 0.71	15 ± 0	17 ± 0
I5	20 ± 0	16 ± 0	17 ± 1.41	17 ± 1.41	16 ± 0	19 ± 1.41
I6	18 ± 0	17 ± 0	16 ± 1.41	16.5 ± 2.12	17 ± 0	17.5 ± 4.95
I8	18.5 ± 0.71	15.5 ± 2.12	16 ± 1.41	17 ± 1.41	16 ± 0	20 ± 4.24
I9	17 ± 0	14.5 ± 0.71	17 ± 0	15.5 ± 0.71	14.5 ± 0.71	15.5 ± 3.54
I12	18.5 ± 0.71	15 ± 2.83	18.5 ± 0.71	16.5 ± 0.71	15.5 ± 0.71	17 ± 4.24
I13	19 ± 0	15.5 ± 0.71	17 ± 0	17 ± 0	15 ± 0	17.5 ± 0.71
c1	18.5 ± 2.12	14.5 ± 0.71	15.5 ± 0.71	15 ± 0	15.5 ± 0.71	14 ± 0
c2	18.5 ± 0.71	15 ± 1.41	15.5 ± 0.71	16 ± 1.41	15.5 ± 0.71	14.5 ± 0.71
c3	19.5 ± 0.71	14.5 ± 0.71	15 ± 0	14.5 ± 0.71	15.5 ± 0.71	14 ± 0
c5	18.5 ± 2.12	17 ± 1.41	17 ± 1.41	15 ± 0	14.5 ± 2.12	15.5 ± 2.12
c9	20 ± 0	14 ± 1.41	16 ± 1.41	17 ± 0	14.5 ± 0.71	15 ± 1.41
c12	17.5 ± 0.71	14 ± 1.4	16.5 ± 0.71	15.5 ± 0.71	15 ± 0	14 ± 0
c13	17 ± 1.41	11.5 ± 2.12	15.5 ± 2.12	16.5 ± 0.71	12.5 ± 0.71	14 ± 1.41
c14	17.5 ± 0.71	14 ± 1.41	17 ± 1.41	14.5 ± 0.71	14 ± 1.41	15 ± 0
c19	20 ± 0	13.5 ± 0.71	18 ± 1.41	13.5 ± 0.71	14.5 ± 0.71	15 ± 1.41

Data are mean values ± SD of independent experiments (*n* = 3)

#### Agar spot test

As in the agar well diffusion assay, only 18 LAB isolates inhibited all the pathogens, with zones of inhibition ranging from 1 to 4.0 mm (with the exception of LAB strain c13, which showed no activity to *E. coli* O157: H7 and *E. faecalis*). Although, pathogen inhibition by the LAB strains is strain and pathogen specific, LAB strains isolated from the intestine showed greater activity against the tested pathogens than the LAB strains isolated from the crop. Also, LAB strains examined showed wider inhibition zones against *E. coli, L. monocytogenes* and *E. faecalis* than against *S*. Enteritidis respectively (Table 2). LAB isolates that failed to show antimicrobial activity against the pathogens examined were immediately discontinued from preceding evaluation.

**Table 2:** Antagonistic activity of potential lactic acid bacteria (LAB) probiotic strains from poultry against pathogenic bacteria by agar spot test

			Inhibition ± SD^a^			
Strain	*Escherichia coli*	*Escherichia coli* O157:H7	*Enterococcus faecalis*	*Salmonella* Typhimurium	*Salmonella* Enteritidis	*Listeria monocytogenes*
I1	3 ± 0	3 ± 0	2.3 ± 0.6	2 ± 0	2 ± 0	2 ± 0
I2	3.3 ± 0.6	2 ± 0	2.5 ± 0.4	1.8 ± 0.2	1 ± 0	2 ± 0
I4	2 ± 0	1 ± 0	1.7 ± 0.7	1 ± 0	1 ± 0	2 ± 0
I5	4 ± 0	2 ± 0	3 ± 0.1	3 ± 0	2.1 ± 0.5	3 ± 1
I6	2 ± 0	2 ± 0	2.3 ± 0.7	2 ± 0	2 ± 0	2 ± 0
I8	3 ± 0.1	2 ± 0	2 ± 0.3	2 ± 1	2 ± 0	3 ± 0
I9	2 ± 0	1 ± 0.5	2 ± 0	1.5 ± 0.4	1 ± 0	1 ± 0
I12	1 ± 0	1 ± 1	1.7 ± 0.4	1.5 ± 0.1	1 ± 0	2 ± 0
I13	3.2 ± 0.5	2 ± 0	2 ± 1	2.7 ± 0.6	1.7 ± 0.2	2.3 ± 0.5
c1	2 ± 0.1	1 ± 0.7	1.7 ± 0.3	1.5 ± 0	1 ± 0.5	1 ± 0
c2	2 ± 0.5	1 ± 0	1 ± 0	1.3 ± 0.4	1 ± 0.2	1 ± 0
c3	3 ± 0.1	2 ± 0	2.1 ± 0.5	1 ± 0	2 ± 0	2 ± 0.3
c5	2.4 ± 1	2 ± 0.3	2 ± 0.8	1.3 ± 0.3	1 ± 0.1	2 ± 0.7
c9	3 ± 0	2 ± 1	2 ± 0	2 ± 0	1.7 ± 0.6	2 ± 0
c12	2 ± 0.2	1 ± 1	1.4 ± 0.3	1.5 ± 0.9	2 ± 0	2 ± 0.4
c13	2 ± 0.1	0 ± 0	1 ± 0	1 ± 0.8	1 ± 0.3	1 ± 0.2
c14	3 ± 0	1.7 ± 1	2 ± 0	1.3 ± 0.3	1 ± 0	1.4 ± 0.3
c19	4 ± 0	2 ± 0	2.7 ± 1	1 ± 0	1.6 ± 0.3	1 ± 1

^a^Values are expressed as the mean ± SD of triplicate independent experiments

### Safety of LAB probiotic strains

#### LAB Haemolytic ability

Three (3) out of the 18 LAB isolates examined for haemolytic activity were β-haemolytic and so were screened out of the subsequent screening for probiotic properties, as they are not considered safe. As such, only 15 LAB isolates (7 and 8 from intestine and crop) were used for subsequent assays (Fig. 1).

#### Bile salt tolerance

All the 15 LAB isolates (7 and 8 from intestine and crop) tested show good tolerance to 0.3% bile salt after 6 h of exposure. No statistical difference (*P* > 0.05) in the LAB strains viability after 3 and 6 h of incubation. Furthermore, the range of intestine LAB strains viability (Log_10_ CFU ml^− 1^) after 6 h incubation was between 8.925 ± 0.055 to 9.245 ± 0.077 while 8.847 ± 0.048 to 9.131 ± 0.029 was recorded for LAB strains from the crop (Table 3).

**Table 3:** Potential lactic acid bacteria (LAB) probiotic strains viability (Log_10_ CFU ml^− 1^) after 0, 3 and 6 h incubation in 0.3% bile salt

	0.3% Bile Salt		
Strains	0 h	3 h	6 h
I1	8.938 ± 0.088	8.914 ± 0.064	8.878 ± 0.012
I2	9.107 ± 0.122	9.094 ± 0.115	9.245 ± 0.077
I4	8.967 ± 0.083	9.160 ± 0.190	9.025 ± 0.017
I5	9.027 ± 0.199	9.031 ± 0.197	9.110 ± 0.014
I8	8.972 ± 0.075	8.911 ± 0.019	8.925 ± 0.055
I9	8.944 ± 0.066	8.934 ± 0.075	8.947 ± 0.069
I13	8.979 ± 0.035	8.986 ± 0.038	8.981 ± 0.045
c1	8.911 ± 0.019	9.037 ± 0.189	8.966 ± 0.066
c2	8.951 ± 0.074	8.916 ± 0.011	8.908 ± 0
c3	8.936 ± 0.078	8.895 ± 0.020	8.895 ± 0.012
c5	9.011 ± 0.015	9.033 ± 0.023	9.131 ± 0.029
c9	8.931 ± 0.032	8.914 ± 0.022	8.949 ± 0.007
c13	9.027 ± 0.199	8.869 ± 0.033	8.902 ± 0.038
c14	9.028 ± 0.154	9.082 ± 0.086	9.100 ± 0.066
c19	8.926 ± 0.141	8.905 ± 0.170	8.847 ± 0.048

Values are means of duplicate experiments; ± indicates standard deviation from the mean. Values are not significant different (*P* > 0.05)

#### Simulated gastric juice survivability with and without lysozyme (pH 2)

**Fig. 2:**
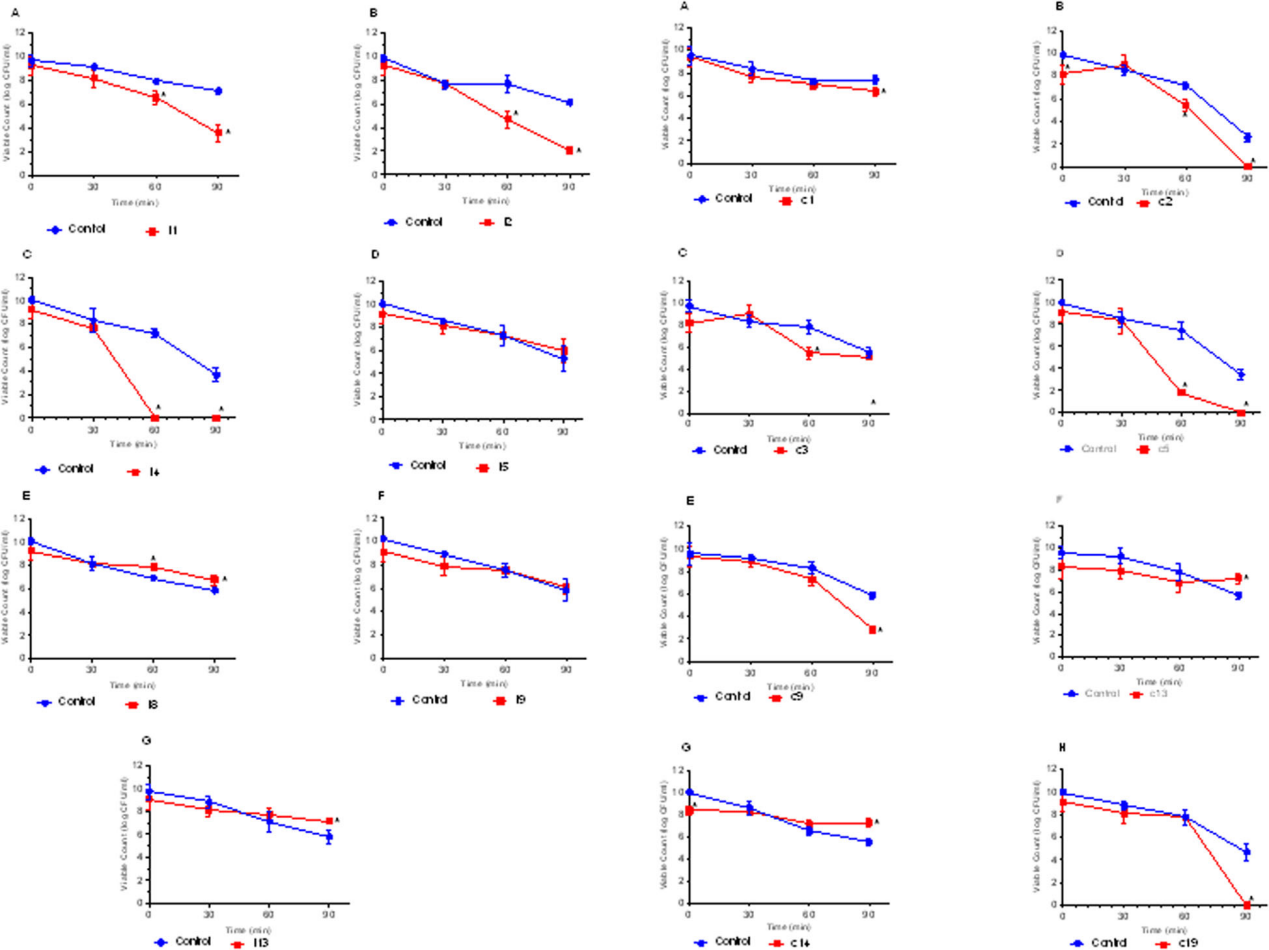
Survival of lactic acid bacteria (LAB) strains from poultry crop in stimulated gastric juice pH 2.0. The data are the means of triplicate experiments, and error bars indicate SD. *values are significantly different (*P* < 0.05). A-H are LAB strains examined

LAB isolates survivability in simulated gastric juice with lysozyme at pH 2.0 was examined. After 90 min exposure to the simulated environment, only 6 LAB strains isolated from the intestine were able to survive with viable counts > 2.0 CFU/ml. There were significant differences (*P* < 0.05) in the viability of LAB strains I1, I2 and I13 with the control after 90 min of incubation. Also, only 5 of the LAB strains isolated from crop were able to survive the simulated environment after 90 min of incubation, with viable counts > 3.0 CFU/ml. LAB strains c1, c9, c13 and c14 showed significant differences (*P* < 0.05) in their viability with the control after 90 min of incubation while c3 showed no difference (*P* > 0.05) with the control (Fig. 2).

#### Adhesion of LAB strains to chicken ileum epithelial cells

**Fig. 3:**
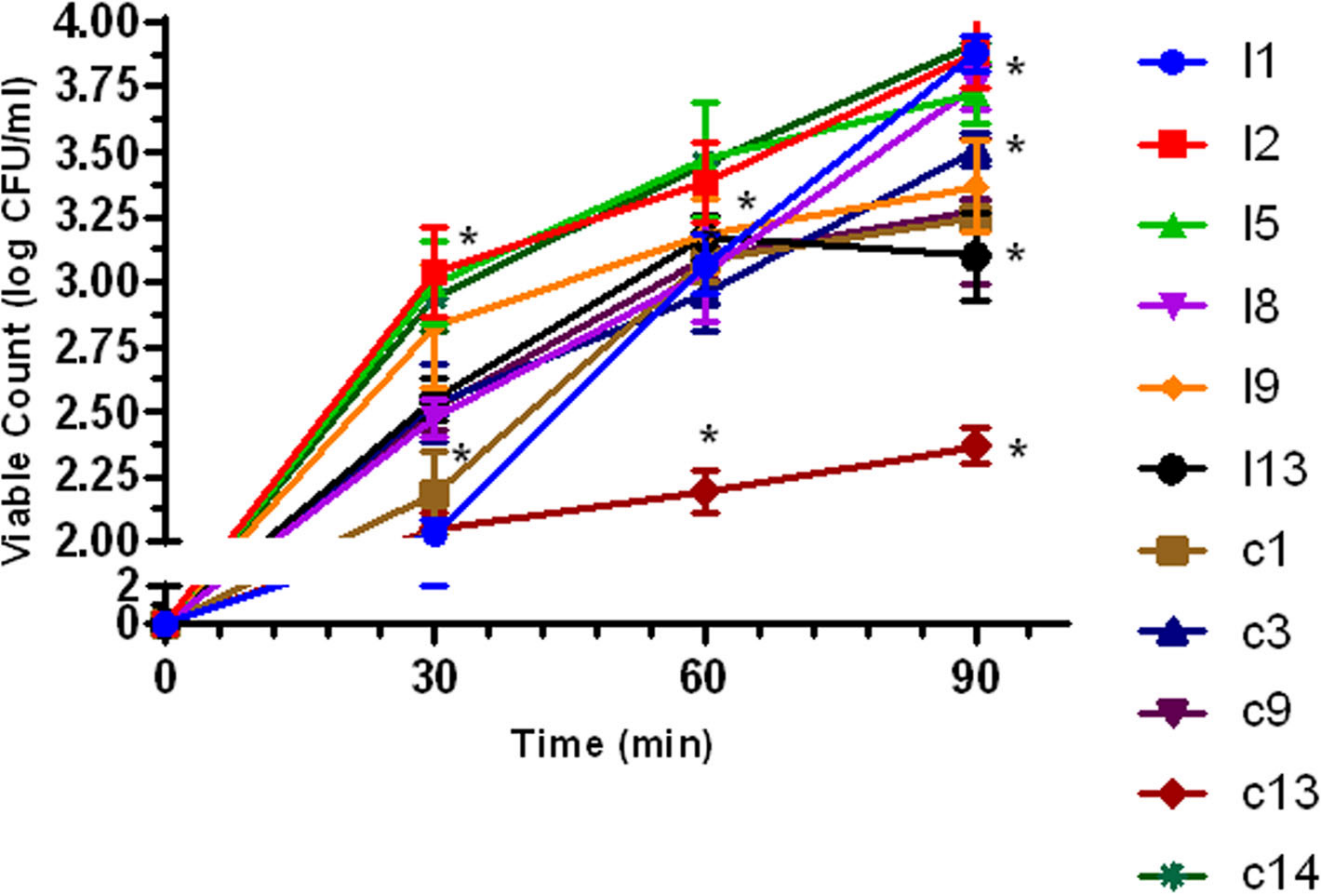
Adherence of poultry lactic acid bacteria (LAB) strains to poultry ileum epithelial cell. The data are the means of triplicate experiments, and error bars indicate standard deviations. *values are significantly different (*P* < 0.05)

The ability of LAB Strains to adhere to chicken ileum epithelial cells was assessed. All the LAB strains adhere to the epithelial cells with a gradual increase in their viability count from time 0 to 90 min of incubation. Multiple comparison test of the adherence capability shows a statistically significant difference (*P* < 0.05) between the LAB strains viability counts after 30, 60 and 90 min incubation (Fig. 3).

#### Phenol tolerance

**Fig. 4:**
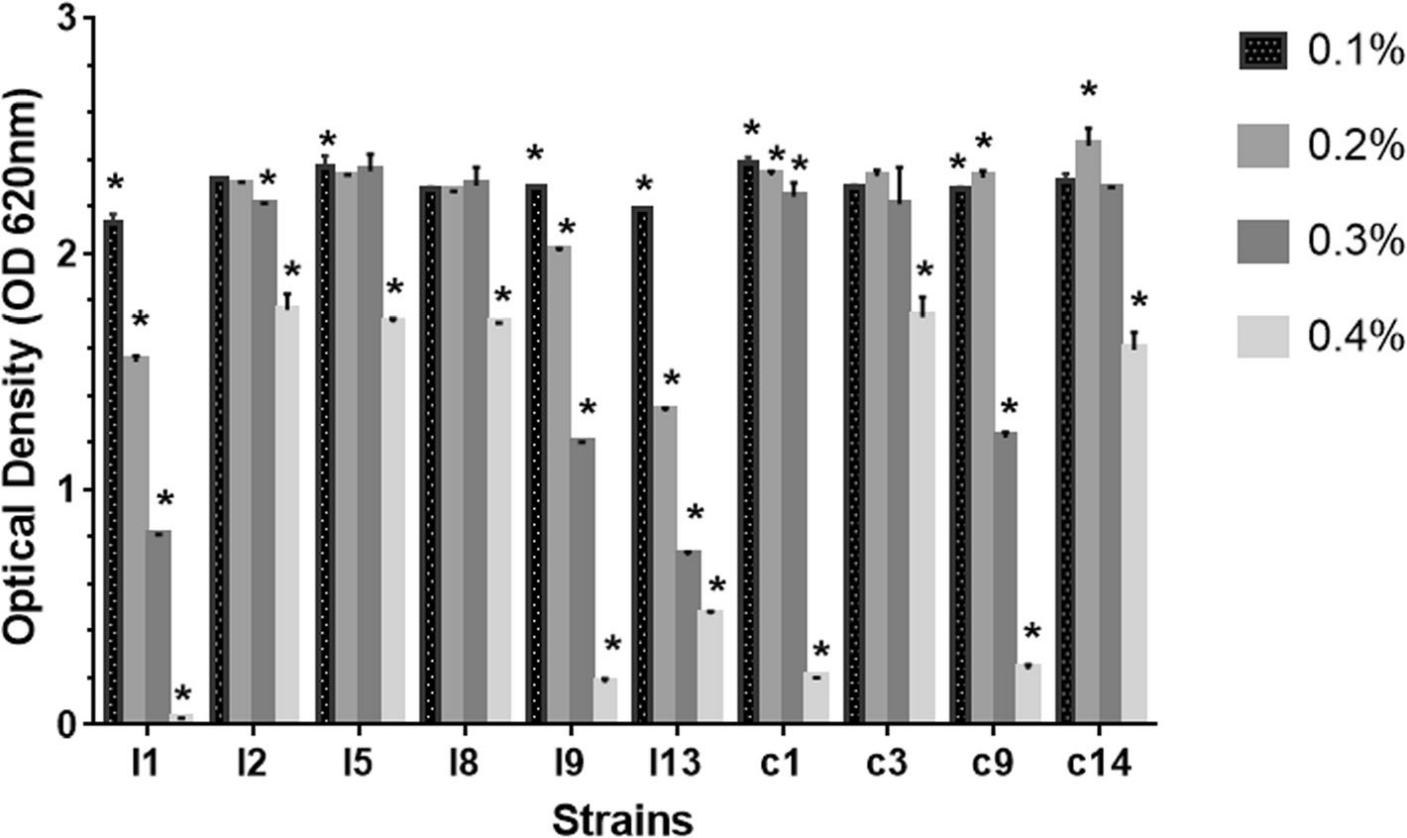
Tolerance to 0.10, 0.20, 0.30 and 0.40% phenol by lactic acid bacteria (LAB) strains from poultry. The data are the means of triplicate experiments, and error bars indicate standard deviations. *values are significantly different (*P* < 0.05)

The result of the LAB isolates tolerance to 0.1–0.4% phenol concentration is shown in Fig. 4. Only 6 (4 and 2 from intestine and crop) LAB isolates were able to tolerate 0.4% phenol with OD values > 1.000. The viability of all the LAB isolates examined differ significantly (*P* < 0.05) with respect to phenol concentration.

#### Temperature and NaCl tolerance

All the LAB isolates grew optimally at 37 °C after 24 h incubation (Fig. 1). Nevertheless, at 4 °C and 55 °C growths were drastically reduced for all the isolates examined. Furthermore, all the LAB isolates tolerated increasing NaCl concentration to 6.5% with OD > 0.500 except isolate c14 with OD of 0.310. Also, at 10.0% NaCl, there was very weak growth of all the isolates ranging from 0.115 to 0.177.

#### Competitive adherence of LAB strains and pathogens to ileum cells

**Fig. 5:**
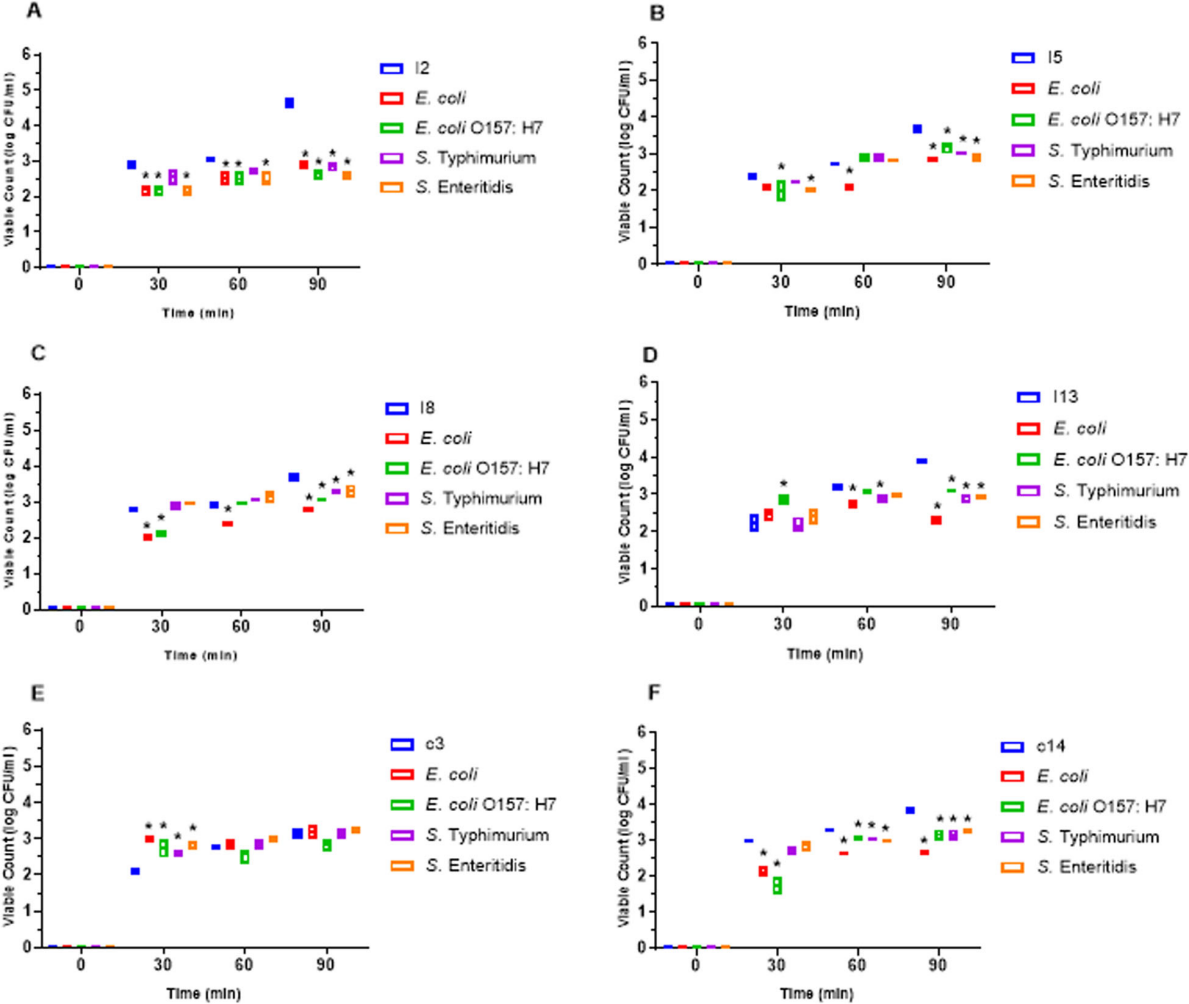
Competitive adherence lactic acid bacteria (LAB) strains and pathogens to poultry ileum epithelial cell. The data are the means of triplicate experiments, and error bars indicate standard deviations. *values are significantly different (*P* < 0.05). A-F are different LAB strains examined

The ability of LAB Strains to competitively adhere to chicken ileum epithelial cells while excluding pathogens was assessed. LAB strains from intestine were able to adhere to ileum cells while successfully excluding all the pathogens tested. After 90 min incubation, there were significant differences (*P* < 0.05) between the viable count of all the LAB strains and the pathogens examined. Whereas the viable count of the entire LAB strains examined ranges between 3.65 ± 0.11 to 4.63 ± 0.14 Log CFU/ml (except LAB strain c3 which had 3.14 ± 0.14) after 90 min of incubation, the viable count of all the pathogens was between 2.30 ± 0.10 to 3.27 ± 0.06. The adhesion of all LAB strains to the ileum epithelial cells differed significantly (*P* < 0.05) from that of all the pathogens after 90 min of incubation. Nevertheless, there was no statistically significant difference (*P* < 0.05) between the adhesion of LAB c3 with all the pathogens examined after 90 min of incubation (Fig. 5).

### Cell surface characteristics

#### Auto-aggregation and co-aggregation ability

Table 4 shows the results of both auto-aggregation and co-aggregation abilities of all the LAB strains examined. The auto-aggregation of these isolates ranged from 32 ± 5.66 to 56.5 ± 3.54%. The auto-aggregation of LAB strains I5 and I13 differ significantly (*P* < 0.05) from other strains respectively. Although the co-aggregation abilities of these LAB strains as revealed from this study is strain specific, and also dependent on the pathogen tested, all the LAB examined show co-aggregation abilities to all the tested pathogens. Generally, the co-aggregation was between 24.03 ± 0.04 (*E. faecalis*) to 83.6 ± 0.83% (*L. monocytogenes*). The co-aggregation abilities of all the LAB strains to *E. coli* O157: H7 differs significantly (*P* < 0.05). Also, high co-aggregation abilities were recorded for LAB strains I2 and c3 while I5 had the least values.

**Table 4:** Aggregation abilities of potential lactic acid bacteria (LAB) probiotic strains from poultry

Co-aggregation (%)							
Strain	Auto-aggregation (%)	*Escherichia coli*	*Escherichia coli* O157: H7	*Enterococcus faecalis*	*Salmonella* Typhimurium	*Salmonella* Enteritidis	*Listeria monocytogenes*
I2	52.50 ± 0.71	75.81 ± 0.98*	83.07 ± 0.08*	68.49 ± 0.6*	66.30 ± 0.39*	66.66 ± 1.0*	71.83 ± 1.82*
I5	32 ± 5.66*	42.46 ± 0.64*	49.04 ± 1.02*	44.01 ± 1.39	49.7 ± 0.59	38.78 ± 0.33	50.81 ± 0.37
I8	47 ± 0	62.38 ± 2.05*	65.45 ± 2.04*	53.82 ± 2.7*	53.76 ± 0.37*	42.75 ± 2.33	60.49 ± 0.04*
I13	56.5 ± 3.54*	53.92 ± 0.02	64.22 ± 0.02*	47.48 ± 0.5*	53.25 ± 1.13*	50.42 ± 1.9*	56.98 ± 2.79
c3	51 ± 0	71.03 ± 1.59*	33.4 ± 0.57*	64.26 ± 0.2*	78.51 ± 0.59*	68.02 ± 0.0*	83.6 ± 0.83*
c14	40.5 ± 3.54	60.01 ± 1.31*	46.68 ± 0.74*	24.03 ± 0.0*	45.46 ± 0.83*	36.16 ± 1.6*	48.03 ± 4.82

Data are mean ± SD of results from triplicate experiments. *values are significantly different (*P* < 0.05)

#### Cell surface hydrophobicity

**Fig. 6:**
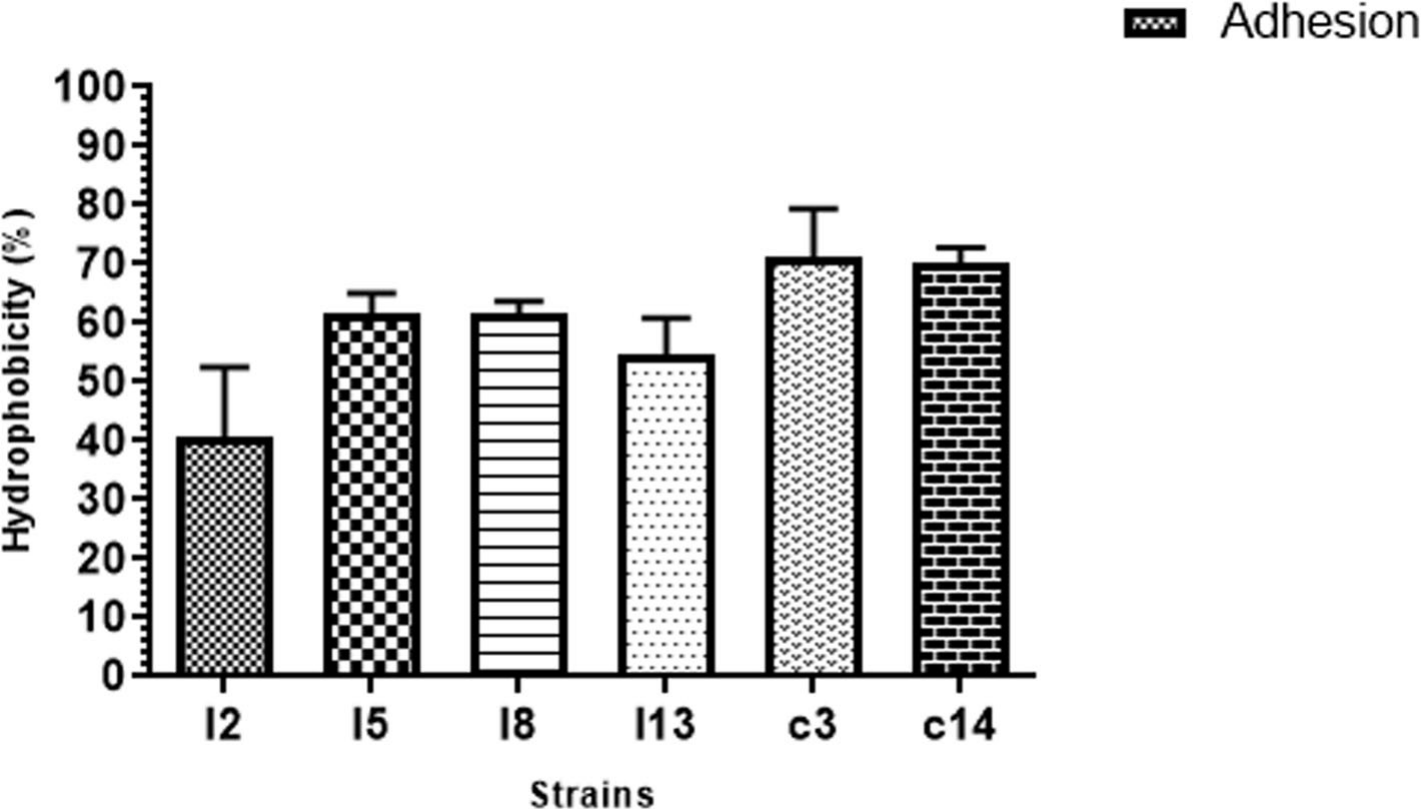
Adhesion of poultry lactic acid bacteria (LAB) strains to n- hexadecane. Results are expressed as mean ± SD of triplicate experiments. Values without * are not significantly different (*P* > 0.05)

The results of the cell surface hydrophobicity of the LAB strains examined is shown in Fig. 6. Although the mean of the values of hydrophobicity obtained for the LAB strains do not differ significantly (*P* > 0.05), isolates from crop tend to have higher values (70.0 ± 2.84 to71.0 ± 8.48) than those from the intestine (with values ranging from 40.5 ± 12.02 to 61.5 ± 3.54).

##### α–Glucosidase inhibitory activity of LAB strains

**Fig. 7:**
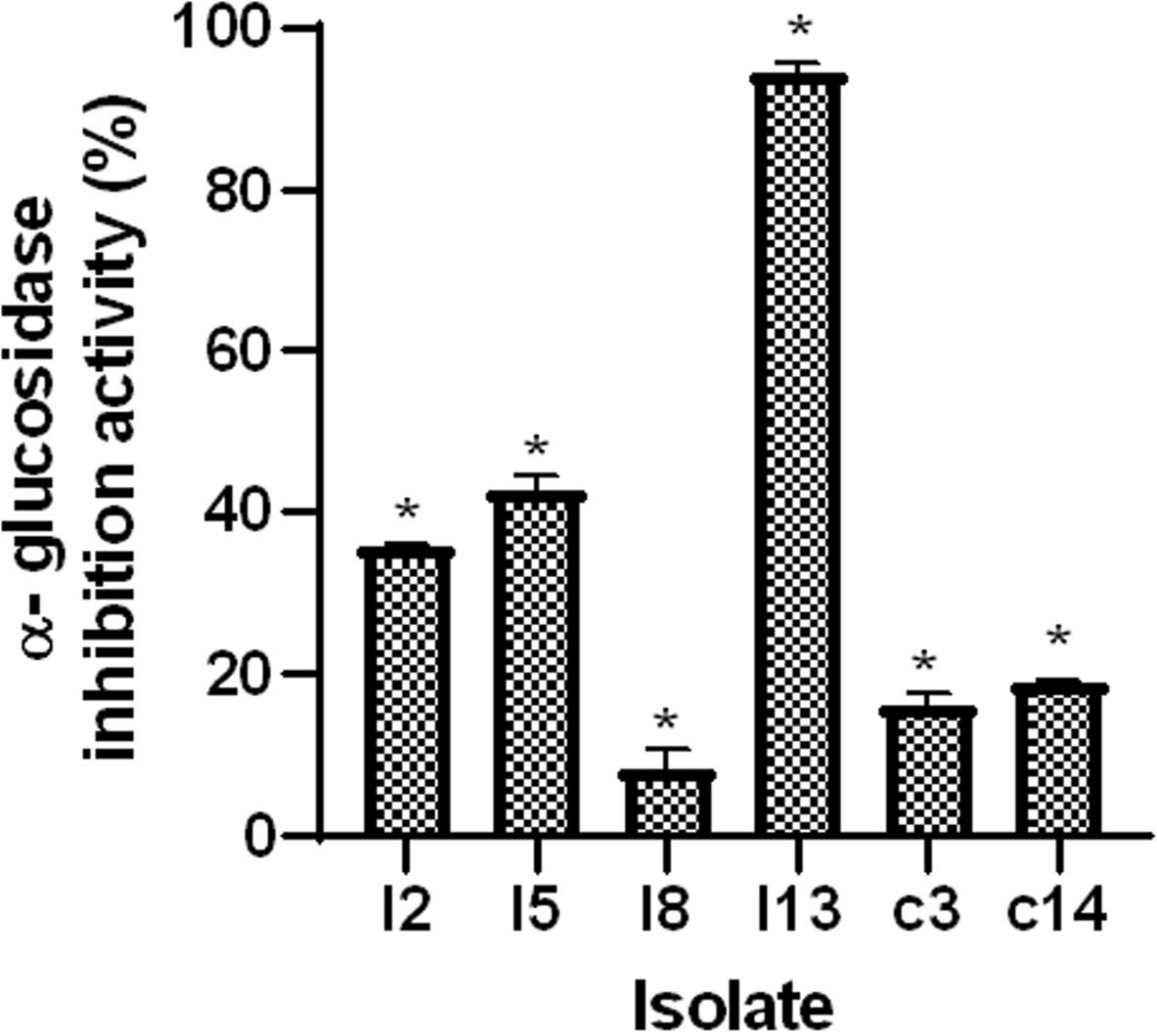
α-glucosidase inhibitory activity of lactic acid bacteria (LAB) strains from poultry. Data are expressed as mean expressed as mean ± SD from 3 independent experiments. *significant differences at *P* < 0.05

The α-glucosidase inhibitory activity results of potential LAB probiotic strains is shown in Fig. 7, with values ranging from 8.44 to 94.41%. All LAB probiotic strains from both crop and intestine showed significant difference (*P* < 0.05) when compared with the positive control.

#### Characterization of LAB antimicrobial substances

Potential LAB probiotic strains were characterized for the production of inhibitory substances such as bacteriocin, hydrogen peroxide and organic acid. Untreated and heat treated (100 °C) CFS show wide zones of pathogens growth inhibition. The antimicrobial substances produced by LAB strains examined were heat stable. Nevertheless, there was reduced (for LAB strains I8 and I13) and complete loss (for LAB strains I2, I5, c3 and c14) of antimicrobial activity by neutralized (pH 7) CFS of LAB cultures (Table 8). This indicated that acid production contributed hugely to the inhibitory effect of these LAB isolates and the effects of bacteriocins as seen in some strains. Furthermore, supernatants that were heat treated did not also affect pathogens inhibition. This implies that heat-labile component produced by LAB strains were not responsible for the inhibition of pathogens. Catalase treated supernatants of LAB strains showed no effect on the inhibitory activities of the six LAB strains examined. This depicts that microbial inhibitory activity by these LAB strains was not as a result of the production of hydrogen peroxides.

#### Antibiotic susceptibility

The antibiotic susceptibility profile of the LAB isolates to 12 commonly used antibiotics was examined (Table 5). Exactly, 100.00% resistance was shown by all the 6 LAB isolates to oxacillin and 83.33% resistance by 5 isolates to erythromycin, vancomycin, ciprofloxacin, streptomycin and tetracycline. All the 6 isolates (100.00%) were susceptible to penicillin, as 5 (83.33%) were susceptible to chloramphenicol while 4 (66.67%) to ceftriaxone and ampicillin respectively (Table 6). Four different resistant phenotypes were expressed by these strains of LAB examined. Strains I2 and I5, and c3 and c14 showed the same resistant phenotype consisting of Erythromycin-Oxacillin-Vancomycin-Ciprofloxacin-Streptomycin-Tetracyclin, and Ceftriaxone-Erythromycin-Oxacillin-Vancomycin-Ciprofloxacin-Streptomycin-Tetracyclin-Gentamycin while strain I8 and I13 also expressed different resistant phenotypes comprising only Oxacillin, and Erythromycin-Oxacillin-Vancomycin-Ciprofloxacin-Streptomycin-Tetracyclin-Gentamycin respectively. Generally, isolates from the crop were resistant to more antibiotics than those from the intestine. The multiple antibiotic resistance (MAR) indexes of LAB strains examined all were above 0.2 except for strain I8 with 0.08. The MAR of isolates obtained from the crop was 0.67 while those of isolates obtained from the intestine ranged between 0.5 to 0.58 respectively (Table 7).

**Table 5:** Antibiotic susceptibility profile of potential lactic acid bacteria (LAB) probiotic strains from poultry

Antibiotic Susceptibility^a^												
Strain	CTR	AMP	P	E	OX	NV	VA	C	CIP	S	TE	GEN
I2	S	S	S	R	R	I	R	S	R	R	R	I
I5	S	S	S	R	R	S	R	S	R	R	R	I
I8	S	S	S	S	R	S	S	S	S	I	S	S
I13	S	S	S	R	R	S	R	I	R	R	R	R
c3	R	I	S	R	R	I	R	S	R	R	R	R
c14	R	I	S	R	R	S	R	S	R	R	R	R

*CTR* ceftriaxone, *AMP* ampicillin, *P* penicillin G, *E* erythromycin, *OX* oxacillin, *NV* novobiocin, *VA* vancomycin, *C* chloramphenicol, *CIP* ciprofloxacin, *S* streptomycin, *TE* tetracycline and *GEN* gentamicin

^a^*R* Resistance, *S* Sensitive and *I* Intermediate

**Table 6:** Percentage of Antibiotic susceptibility of potential lactic acid bacteria (LAB) probiotic strains from poultry

Susceptibility (*n* = 6)			
	R	I	S
Antibiotic	No. (%)	No. (%)	No. (%)
Ceftriaxone	2 (33.33)	0 (0.00)	4 (66.67)
Ampicillin	0 (0.00)	2 (33.33)	4 (66.67)
Penicillin	0 (0.00)	0 (0.00)	6 (100.00)
Erythromycin	5 (83.33)	0 (0.00)	1 (16.67)
Oxacillin	6 (100.00)	0 (0.00)	0 (0.00)
Novobiocin	0 (0.00)	2 (33.33)	4 (66.67)
Vancomycin	5 (83.33)	0 (0.00)	1 (16.67)
Chloramphenicol	0 (0.00)	1 (16.67)	5 (83.33)
Ciprofloxacin	5 (83.33)	0 (0.00)	1 (16.67)
Streptomycin	5 (83.33)	1 (16.67)	0 (0.00)
Tetracycline	5 (83.33)	0 (0.00)	1 (16.67)
Gentamicin	3 (50.00)	2 (33.33)	1 (16.67)

*R* Resistant; *I* Intermediate; *S* Sensitive

**Table 7:** Multiple Antibiotic Resistance indices of potential lactic acid bacteria (LAB) probiotic strains from poultry

Strain	No. of Antibiotic Resistant	MAR Indices
I2	6	0.5
I5	6	0.5
I8	1	0.08
I13	7	0.58
c3	8	0.67
c14	8	0.67

*MAR* Multiple antibiotic resistance

#### Biochemical identification of LAB strains using API 50 CHL

The carbohydrates fermentation profile of all the LAB strains were determined by API 50 CHL micro-identification system8. The isolates showed varying results of their reaction with the 50 substrates tested. For the identification of each LAB isolate examined, the results of their reactions were inputted into the apiweb™ Software version 5.0 (BioMèrieux, France, and the identity of each strain was obtained (Table 9).

#### Molecular identification of LAB strains

**Fig. 8:**
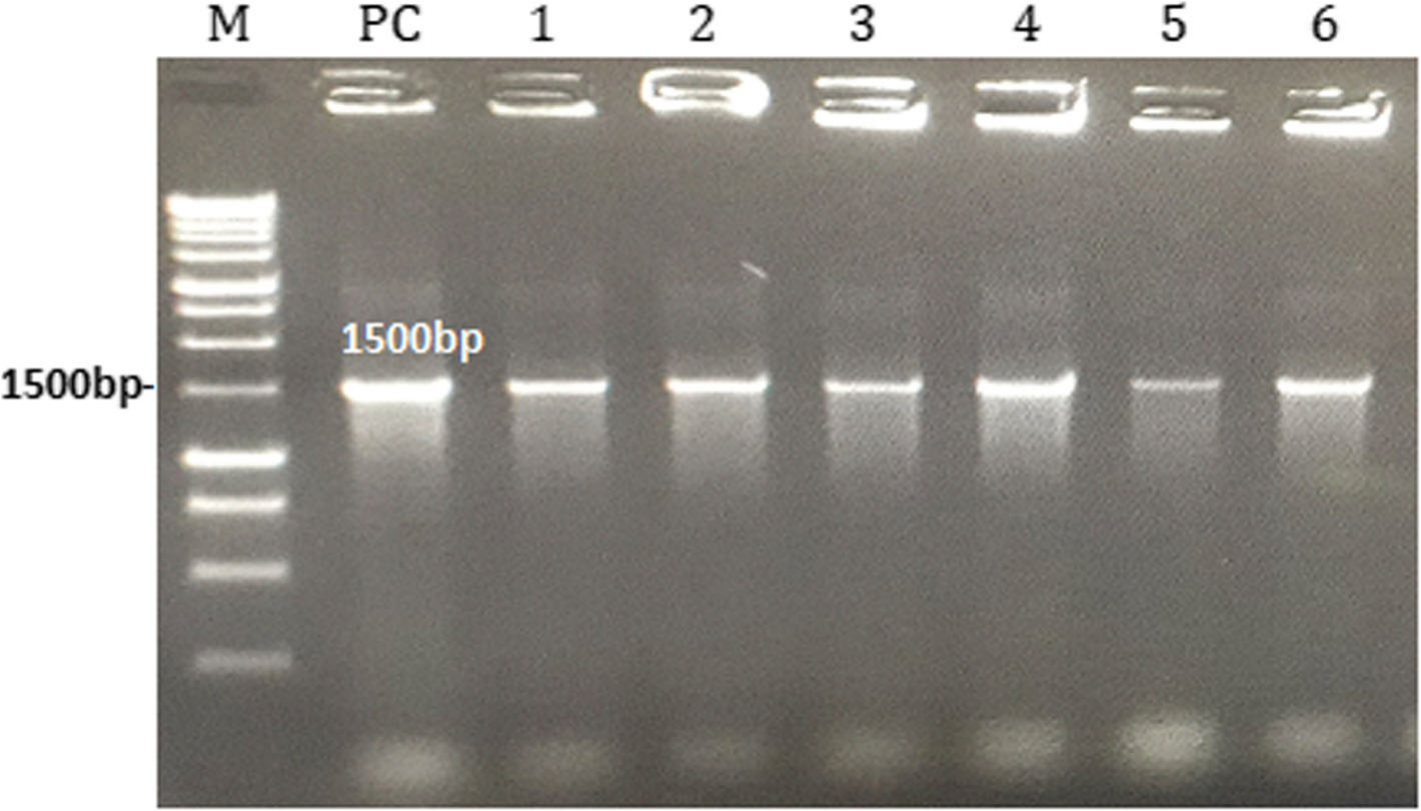
Agarose gel electrophoresis of PCR products after amplification of 16S rRNA; lane M, 1 kb Marker (PROMEGA, USA), lane PC, Positive control (*Lactobacillus casei* ATCC 393), Lanes 1, 2, 3, 4, 5 and 6 are positive lactic acid bacteria (LAB) strains I2, I5, I8, I13, c3 and c14 at 1500 bp

**Fig. 9:**
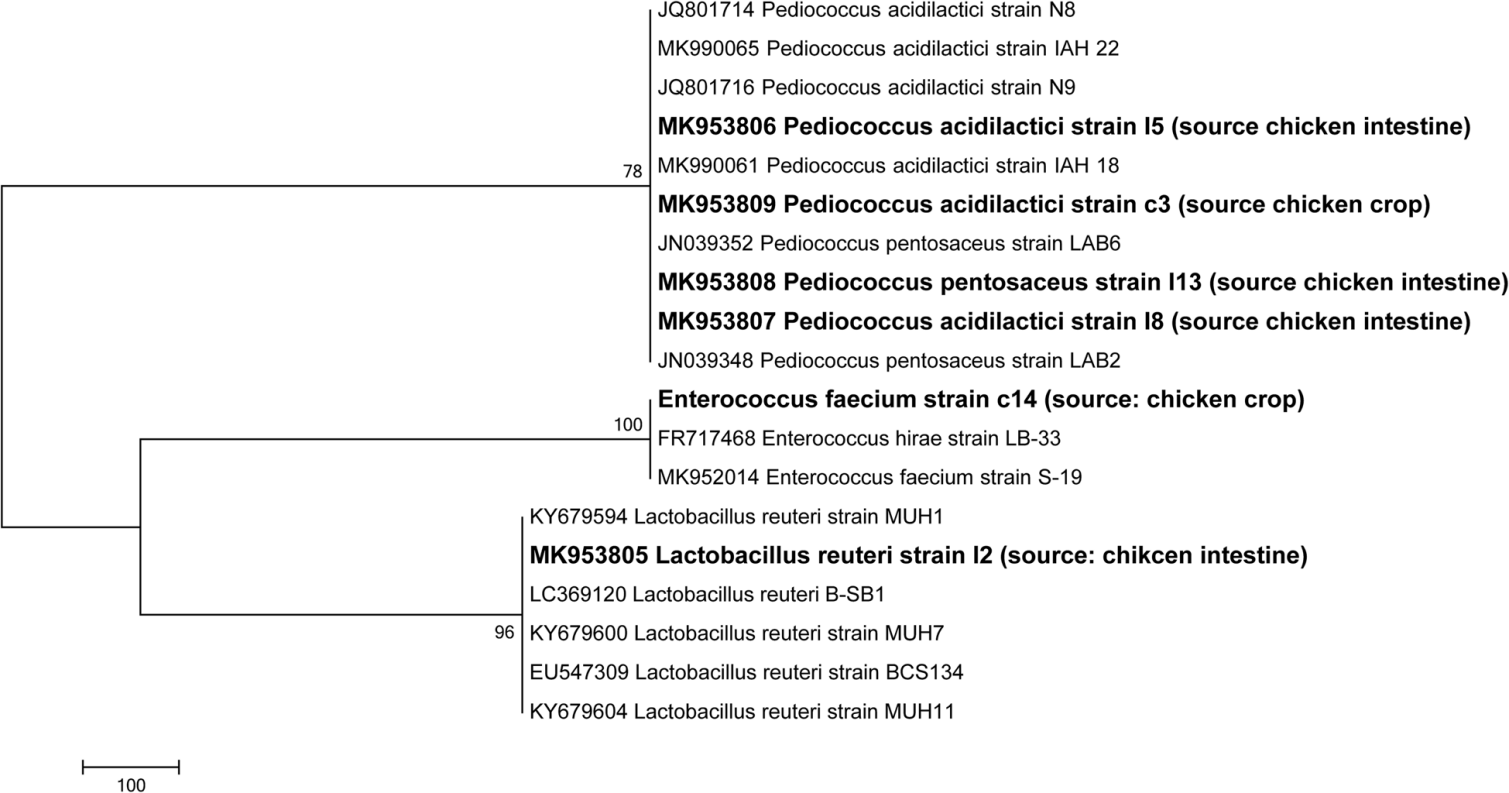
Phylogenic tree based on 16S rRNA gene sequences of potential lactic acid bacteria (LAB) strains in relation to the isolates and type strains. The isolates sequenced in the study are depicted in bold font mentioning the accession number and the source of the samples within bracket. Four strains are under the *Pediococcus* genus, one is *Enterococcus faecium* and another is *Lactobacillus reuteri*

Potential LAB probiotics strains were identified using the 16S rRNA sequencing. All the 6 LAB strains examined were positive with 1500 bp size after agarose gel electrophoresis of amplified PCR products (Fig. 8). The 16S rRNA sequences obtained were blasted and finally deposited in GenBank (https://www.ncbi.nlm. nih.gov/genbank/) under accession numbers (Table 8). Furthermore, Fig. 9 shows the Phylogenic tree based on 16S rRNA gene sequences of potential LAB strains in relation to the isolates and type strains. The sequences of LAB strains analyzed aligned with the 16S DNA sequences as obtained from the database of the Gen-Bank. The 6 LAB isolates were identified as: *Lactobacillus reuteri* I2, *P. acidilactici* I5, *P. acidilactici* I8, *P. pentosaceus* I13, *P. acidilactici* c3 and *Enterococcus faecium* c14.

**Table 8:** The Effects of pH and heat treatment on antimicrobial activity of potential lactic acid bacteria (LAB) probiotic strains

Treatment	Indicator Strain		Inhibition zone by					
			I2	I5	I8	I13	c3	c14
Untreated	*E. coli*		+++	+++	++	++	+++	++
	*E. coli* O157: H7		++	++	++	++	++	++
	*E. faecalis*		++	++	++	++	++	++
	*S*. Typhimurium		++	++	++	++	+	+
	*S*. Enteritidis		+	++	++	++	++	+
	*L. monocytogenes*		++	++	+++	++	+	++
Neutralized	*E. coli*		––	––	+	++	––	––
	*E. coli* O157: H7		––	––	––	+	––	––
	*E. faecalis*		––	––	+	++	––	––
	*S*. Typhimurium		––	––	––	+	––	––
	*S*. Enteritidis		––	––	––	+	––	––
	*L. monocytogenes*		––	––	––	––	––	––
Heat treated	*E. coli*		++	+++	++	++	+++	+++
	*E. coli* O157: H7		++	++	++	++	++	+++
	*E. faecalis*		++	++	++	++	++	+++
	*S*. Typhimurium		++	++	++	++	+	+++
	*S*. Enteritidis		+	++	++	++	++	+++
	*L. monocytogenes*		++	++	+++	++	++	+++
Catalase treated	*E. coli*		+++	+++	++	++	+++	++
	*E. coli* O157: H7		++	++	++	++	++	++
	*E. faecalis*		++	++	++	++	++	++
	*S*. Typhimurium		++	++	++	++	+	+
	*S*. Enteritidis		+	++	++	++	++	+
	*L. monocytogenes*		++	++	+++	++	+	++

Symbols show zones of inhibition (mm): ––, no inhibition; +, weak (< 14); ++, good (15–19); +++, strong (> 20), Neutralized; supernatant treated with 6 N NaOH to obtained a pH 7, heat treated; Supernatants boiled for 10 min, Catalase treated; supernatant treated with 0.5 mg/ml catalase

## Discussion

The application of probiotics in the poultry industry as suitable alternative to antibiotics as well as for improving the performance and productivity of birds have received tremendous attention in recent years. Apart from other beneficial properties of probiotics, sourcing probiotic strains from their natural host is most preferred, as such microbial strains are already familiar with the GIT, and can spontaneously proliferate and express the desired beneficial effects better than strains isolated from other sources. Therefore, the need to develop host-specific probiotic for optimal health benefits and livestock performance is imperative [14]. Furthermore, direct evaluation of potential probiotics in vivo is often expensive and time-consuming. Consequently, in vitro evaluation as major criteria for probiotic selection is to find the most efficient and suitable strain with optimal beneficial properties. Also, grading the extent of health and beneficial effect(s) expressed by specific potential probiotic strain in vivo can be very difficult and expensive [15].

LAB strains were selectively isolated from broilers using MRS medium with pH ranging between 6.4 ± 0.2 – 6.5 ± 0.2, and then evaluated towards their development as poultry probiotics. The optimal pH for the growth of LAB was reported to range between 6.2 – 8.5 [16]. After isolating 57 strains of LAB from the crop (35) and intestine (22) of broilers as previously described [13], they were subjected to various contemporary in vitro probiotic properties evaluation and finally characterized molecularly.

Infections by zoonotic and foodborne enteric pathogens cause high morbidity and mortality with significant economic loss in the poultry industry [17]. Also, these pathogens are often transmitted to humans either via occupational exposure or through the food chain, which is of immense public health concern. Antimicrobial activity against these pathogens is a major requirement of potential probiotics. Out of the 57 LAB strains examined, 18 (9 each from crop and intestine) showed broad-spectrum antagonistic activity against the six pathogens tested (Table 1). There was no discrepancy in the antagonistic activity shown against Gram negative and Gram positive pathogens by our LAB strains, nevertheless, least inhibitory zones were recorded against *S.* Enteritidis. In agreement with our finding, Shin et al. [18], Taheri et al. [19], Yaneisy et al. [20], Busayo et al. [21], and Olufemi et al. [22] reported antagonistic activity against wide spectrum of pathogens by LAB isolated from poultry. Conversely, Kizerwetter-Swida and Binek [23] reported higher antagonistic activity by strains of LAB against Gram positive pathogens (including *Clostridium perfringens* and *Staphylococcus aureus*) than Gram-negative pathogens (including *E. coli* and *Salmonella).* Nevertheless, no relationship between the degree of LAB antagonistic activity and Gram type of pathogens tested was recorded from the finding of de Almeida Júnior et al. [24]. Previously, Spanggaard et al. [25] stated that pathogen antagonism by probiotics was the major influential factor hindering heterochthonous bacteria to establish in the GIT, and this indicates that a significant contribution to the control pathogens is expected when autochthonous microflora are used as probiotics. Similarly, Jose et al. [26] found that LAB strains from animal rumen inhibited the growth of pathogens better that LAB strains from dairy sources. The specificity of the agar spot test revealed less inhibition zones against pathogens that earlier had wider zones of inhibition as recorded from the agar well diffusion technique. This agrees with the findings of Armas et al. [27] who recently reported similar activities of LAB strain against array of pathogens using the agar spot assay.

According to FAO [8] guidelines, microbial strains to be used as probiotics are recommended to be safe in the host. The selection and application of strains devoid of haemolytic activity as probiotics, depicts their non-virulent nature. Out of the LAB strain examined for haemolytic activity, 16 were non-haemolytic, and so they were selected for subsequent evaluation since they are safe to use as probiotics. Similar results indicating that majority of LAB strains are non-haemolytic have been previously reported [22].

The ability of potential probiotic strains to tolerate or withstand intestinal bile salt is of immense importance to their survival and growth in the GIT, as such, it is a major requirement for probiotic selection [15]. In the chicken GIT, the duodenum and cecum have a total bile salt concentration of 0.175 and 0.008% [28]. However, the average level of 0.3% bile salt has been considered in many studies for bile salt tolerance of potential probiotic LAB [20, 29]. In our studies, all the LAB strains examined were able to tolerate 0.3% bile salt after 6 h incubation (Table 3). This was expected since the LAB strains originated from chicken. Similarly, all the LAB strains isolated from chicken as reported by Shin et al. [18], and Shokryazdan et al. [29] showed good tolerance to 0.3% bile salts.

Apart from the ability of potential probiotics to tolerate bile salts, it is also expected that probiotics should be able to tolerate the acidic environment of the GIT as they pass through to colonize the gut of their host [30]. The secretion of gastric juice with an approximate pH of 2.0 causes the death of most exogenous microbes when ingested into the GIT [31]. Out of the 15 LAB strains evaluated, four (1 from crop and 3 from the intestine) failed to survive in simulated gastric juice with lysozyme (pH of 2.0), with no viable cells after 90 min of incubation (Fig. 2). LAB are known for their ability to tolerate acidic pH [31]. Our finding corresponds with previous studies who reported moderate to good survival of LAB strains isolated from chicken to simulated gastric juice with pH of 2.0 [1, 18, 20, 32]. Similarly, LAB strains including *L. pentosus* and *L. plantarum* isolated from fermented sausages were able to survive acidic environment [33]. Generally, based on the time of feeding, the age and kind of animal, gastric juice pH concentration may vary from 2.0 to 3.5 [34]. Results obtained from our study revealed that the survival of our potential LAB probiotic strains in simulated gastric juice (pH of 2.0) is strain-specific. Furthermore, strains that were able to survive this environment can also be able to transit the harsh condition of chicken gut and attach to intestinal cells while exerting beneficial effects.

Adherence to host’s intestinal cells is a major feature required by probiotics strains for colonization [35]. Beneficial effects exerted by probiotics including antimicrobial activities against pathogens, immune-modulation, cholesterol lowering etc. are only possible with strong adherence to the epithelial cells of the intestine [29, 36]. All the LAB strains evaluated from this study adhered to chicken ileum epithelial cells with a gradual increase in their viability count time 0 to 90 min of incubation (Fig. 3). Nevertheless, strain c13 showed the least adherence ability with viable cell count < 2.5 CFU/cm^2^, and so it was screened out from preceding evaluation. Jose et al. [26] in their work reported better adherence ability exhibited by LAB isolates from animal rumen that dairy isolates. Also, our finding agrees with Nitisinprasert et al. [37] and Setyawardani et al. [38], who reported the adhesion abilities of LAB to intestinal cells. During peristaltic flow and gut contraction, the adhesion of LAB strains to chicken intestinal cells further protects them from frequent removal [39].

Phenol is a microbial toxic metabolite secreted in the GIT as a result of the deamination of some amino acids [40]. It is expected that potential probiotics should be able to tolerate the effect of phenol. With increasing phenol concentration, LAB strains I1, I9, c1 and c9 poorly tolerated 0.4% phenol concentration with OD < 0.3 (Fig. 4), and so they were discontinued. At 0.4% concentration of phenol, Shehata et al. [41] previously observed tolerance of LAB strains at varying degrees.

Findings from our study further revealed that all LAB strain examined grew optimally at 37 °C after 24 h incubation. At 4 °C and 55 °C the growth of LAB examined was reduced. This finding is in agreement with previous report [42]. Also, our LAB strains showed excellently tolerance to 6.5% NaCl concentration but at 10.0% NaCl weak growth of the strains was recorded. LAB from milk and milk products, meats, chicken faeces etc. are able to survive 1.0–9.0% NaCl [41, 43]. These results are desirable features from potential LAB probiotics which could increase bacterial growth and production of beneficial metabolites. Also, these traits exhibited by these LAB strains are of industrial and technological relevance as well as for preservation.

Apart from good adherence to intestinal cells, probiotics should most importantly have the ability to competitively exclude or inhibit pathogens adhering to hosts’ intestinal cells. Although all the LAB strains examined were able to significantly exclude all the pathogens tested, there were variations in the degree of the pathogen exclusion by LAB isolates (Fig. 5). In agreement with our finding, Kos et al. [44] and Dowarah et al. [14] reported a lot of variation among LAB ability to compete with pathogens when co-cultured. Although some probiotics are administered orally, they should be able to competitively exclude harmful organisms once they successfully colonize the host intestinal cells. LAB adhesion is a complex process initiated from the foremost bacterial contact with the cell membrane of the host enterocytes followed by diverse surface interactions [44]. Most LAB produce cell surface proteins which among other functions aid the bacteria to bind with the epithelium of the GIT. This further enables immunoregulation by LAB which is also relevant in the removal of pathogens [36].

Furthermore, we examined LAB cell-binding abilities that is, autoaggreation and coaggregation abilities. These are 2 of the several factors involved in probiotics adhesion in chicken intestinal cells [45]. Whereas autoaggregation is an important requirement for the formation of biofilm which further aid adhesion and colonization of host intestinal cells by probiotics, coaggregation on the other hand enables probiotics to form barrier that is effective in the prevention of enteric pathogens adhesion on intestinal cells [17]. The results of autoaggregation abilities by LAB strains as recorded from our study was between 32 ± 5.66 to 56.5 ± 3.54% (Table 4). After determining the autoagrregation of 332 LAB strains from chickens, Taheri et al. [19] reported that only 62 strains from cecum (18), ileum (22), and crop (22) showed good autoaggregation abilities. Our findings are in consonance with Puniya et al. [46] who recorded LAB autoaggregation ranging between 30.0–76.0% and 48–73.0% respectively. The coaggregation abilities between the potential LAB probiotics and the 6 pathogens tested showed strain-and-pathogen specific coaggregation abilities, ranging between 24.03 ± 0.04 (for *E. faecalis*) to 83.6 ± 0.83% (for *L. monocytogenes*). Findings from the work of Venkatasatyanarayana et al. [17] reported 62.2 ± 1.03% and 35.5 ± 1.32% coaggregation between *L. plantarum* to *E. coli* and *L. monocytogenes*.

Another major requirement to be considered when selecting potential probiotic candidates is the strain surface hydrophobicity. The hydrophobicity of probiotics directly measures their adhesion abilities to cellular lines of the enterocytes [47], which is a much-desired property in probiotics. All the LAB strains examined show good hydrophobicity abilities with values ranging from 40.0–70.0%, with strain from the crop showing higher hydrophobicity ability (Fig. 6). On the basis of superiority, hydrophobicity of 40.0% and above against hexadecane was the standard used by Pringsulaka et al. [48] in the selection of LAB probiotics. Dowarah et al. [14], recorded 15–60.0% hydrophobicity of LAB strains from pigs while Karimi et al. [49] and Yaneisy et al. [20], recorded hydrophobicity of between 3.6 ± 0.19–93.53 ± 3.10 and 30–71.10%, from LAB strains isolated from poultry. Also, Ehrmann et al. [15] obtained high hydrophobicity among LAB strains isolated from poultry. Previous reports have shown that a correlation exists between high hydrophobicity of LAB strains with their attachment to intestinal mucosal and epithelial cells [2, 15, 19]. Due to the strong relationship between aggregation and hydrophobicity abilities of probiotics with their adhesion to GIT epithelial cells, potential LAB probiotics could be evaluated for these 2 characteristics instead of mucus adhesion ability [19].

α-glucosidase inhibitory ability of potential probiotic strains is a valuable functional property which we evaluated in this study. Although α-glucosidase inhibitory activity is strain specific, LAB strain I13 from the intestine showed the highest percentage (92.0) of activity while activity by other strains ranged between 8.3 to 45.0% (Fig. 7). This activity could result from LAB ability to produce exopolysaccharides (EPS) [50]. These LAB strains could reduce the absorption of intestinal carbohydrates.

Antagonistic activity by LAB are sustained by the secretion of different antimicrobial substances including organic acids (lactic, acetic etc), bacteriocins, alcohols, hydrogen peroxide, antimicrobial peptide etc. [17]. The substances responsible for the antagonistic activity by most promising LAB probiotic strains selected as revealed in our study were organic acid and low molecular weight substances. When the pH of the CFS was neutralized, all the LAB lost their antagonistic activity against the pathogens examined except LAB strains I8 and I13 which showed weak and moderate antagonistic activity against *E. coli* and *E. faecalis* (by I8), and *E. coli*, *E. coli* O157: H7, *E. faecalis*, *S*. Typhimurium and *S*. Enteritidis (by I13) (Table 8). Similarly, Gusils et al. [51] reported the complete loss of antagonistic activity by 100 LAB strains isolated from GIT of pigs against pathogens when the pH of CFS was neutralized. LAB strains from poultry also lost their inhibitory action after pH neutralization [49]. Lin and Pan [52] reported constant antimicrobial activity within the pH range from 1.0 to 4.0 but complete loss of activity at 5.0 to 11.0 pH. In the same vein, Blajman et al. [1] reported no zones of inhibition against pathogen tested when the CFS of LAB strains isolated from poultry were adjusted to pH 6.5. Also, when the CFS from our LAB strains were heated at 100 °C for 10 min, there was no loss of antimicrobial activity, depicting that the substance(s) responsible for antimicrobial activity may not be heat sensitive. Furthermore, our study revealed that hydrogen peroxide was not responsible for antagonistic activity by the selected LAB strains as there was no effect when the supernatants of our LAB were treated with catalase. Our findings showed that the antimicrobial activity by our LAB strains was as a result of the secretion of organic acids, bacteriocins or other natural antimicrobial substances. The secretion of bacteriocins by LAB is greatly influenced by temperature, pH, time of incubation and some other environmental factors [53]. Also, it has been reported that optimum bacteriocin secretion is between the pH 4 and 5. Bacteriocins secreted by strains of LAB have attracted unprecedented increased attention in the food industry, medical and veterinary medicine due to their safety [32]. From the last few decades, several new bacteriocins secreted by strains of LAB have been identified, named and characterized [54].

The assessment of antimicrobial susceptibility profile is a major criterion for potential probiotics evaluation. Microbial strains to be considered as probiotics should not serve as antibiotic resistance genes reservoir, which may further be transferred to intestinal pathogens [17]. All the 6 (100.00%) LAB isolates were susceptible to penicillin, 5 (83.33%) were susceptible to chloramphenicol while 4 (66.67%) were susceptible to ceftriaxone, ampicillin, and novobiocin (Tables 5 and 6). Dowarah et al. [14] also reported high susceptibility to penicillin, ampicillin and chloramphenicol by LAB strains isolated from pigs and poultry. Also, Puniya et al. [46], and Anandharaj and Sivasankari [55] showed ceftriaxone and novobiocin susceptibility among LAB strains. It has also been documented that lactobacilli are generally susceptible to ampicillin [56]. Nevertheless, all the 6 (100.00%) LAB strains examined were resistant to oxacillin, 5 (83.33%) were resistant to vancomycin, ciprofloxacin, streptomycin, tetracycline while 3(50.00%) were resistant to gentamicin. It has been reported in literature that strains of LAB are resistant to β-lactam antibiotics including oxacillin, because they harbor of β-lactamase [26, 43]. Also, LAB have been reported to have intrinsic resistant to streptomycin and gentamicin, and vancomycin which are aminoglycosides and glycopeptide [26]. This is as a result of their membrane impermeability. Similarly, high natural resistant to ciprofloxacin as obtained in our work has also been reported by Tang et al. [57]. Jose et al. [26] have previously reported tetracycline resistance from LAB strains isolated from milk, animal rumen and most commercial probiotics. The intrinsic antibiotic resistance nature of LAB probiotics suggests their application for both therapeutic and preventive purposes in the treatment and control of intestinal infections especially when they are simultaneously administered alongside antibiotics [58]. Also, the recovery of GIT microflora can be enhanced such probiotics [46].

The high MAR indices recorded among LAB strains isolated from poultry as recorded in our study show that these strains were obtained from environment where there is misuse of antibiotics. Indeed, this is the case with the study area, where antibiotics are discriminately used as feed additives in poultry. Furtula et al. [59] reported that MAR index above 0.2 depicts that such strain is obtained from environment with free access and abuse of antimicrobial agents.

Results from both API 50 CHL and 16S rRNA sequencing shows inconsistencies except for *Pediococcus acidilactici* (I8) and *P. pentosaceus* (I13) (Table 9). Such inconsistencies were also recorded by Boyd et al. [60] who opined that the identification of lactobacilli using API 50 CHL database can lead to uninterpretable or misidentification results. Also, out of the 7 LAB strains identified by both API 50 CHL and 16S rRNA sequencing, only 2 species *L. salivarius* and *P. acidilactici* matched both identification systems [61]. In some cases, commercial systems used for identification often give correct identification of the genus (as the case with LAB strain I2 in our study) but not adequate enough to identify up to species level. De Vries et al. (2006) further reported that similar physiological profiles are often shown by phylogenetically related LAB species, which makes it inadequate to only rely on biochemical methods for identification. Our results confirm the precision and accuracy of 16S rRNA sequencing in the identification of LAB as previously recommended [61].

**Table 9:** Species identification of potential lactic acid bacteria (LAB) Probiotic strains by API 50 CHL and 16S rRNA Sequencing

	Identification by API 50 CHL			Identification by 16S rRNA sequences		
Isolate	Source	Strains	% Accuracy	Strains	Sequence Accession Number	Sequence Identity %
I2	Intestine	*Lactobacillus paracasei ssp paracasei* 1	98.3	*Lactobacillus reuteri* strain I2	MK953805	99.65
I5	Intestine	*Lactococcus lactis* ssp. *lactis* 1	96.9	*Pediococcus acidilactici s*train I5	MK953806	99.72
I8	Intestine	*Pediococcus acidilactici*	99.9	*Pediococcus acidilactici s*train I8	MK953807	99.72
I13	Intestine	*Pediococcus pentosaceus* 2	99.9	*Pediococcus pentosaceus* strain I13	MK953808	99.44
c3	Crop	*Pediococcus pentosaceus* 2	95.5	*Pediococcus acidilactici* strain c3	MK953809	99.87
c14	Crop	*Lactococcus lactis* ssp. *lactis* 1	92.9	*Enterococcus faecium s*train c14	MK953804	98.06

## Conclusion

In conclusion, 6 LAB strains from poultry were found to possess suitable in vitro probiotic properties, including broad spectrum of antimicrobial activity against zoonotic and foodborne pathogens, good ability to competitively exclude pathogens while adhering to chicken ileum epithelial cells, high cell surface properties, survivability in gastric juice (pH 2), and phenol and bile salt tolerance. The six LAB strains identified by 16S rRNA sequencing to be *Lactobacillus reuteri* BCS134 (I2), *Pediococcus acidilactici* R76 (I5 and c3), *Pediococcus acidilactici* X1 (I8) *Pediococcus pentosaceus* LAB2 (I13), and *Enterococcus faecium* ISMMS VRE 2 (c14). These LAB strains are ideal probiotic candidates which can be used in vivo for both biocontrol of intestinal pathogens and to increase poultry performance.

## Methods

### Isolation and phenotypic characterization of LAB strains

LAB strains were isolated from the GIT (crop and intestine) of apparently healthy male and female broilers from the poultry farms in Jashore, Bangladesh. Briefly, the chickens were euthanized using overdose of isoflurane anesthesia followed by cervical dislocation after which their crops and intestines were aseptically removed, placed in sterile plastic bags, and immediately brought to the laboratory for microbial analysis. All efforts were made to minimize suffering. This euthanasia method was adopted for this study due to its rapidity, efficacy, ease of use and operator safety. Also, it does not have deleterious effects on the birds. After removing the content of each section, 10 g of each of the GIT section was aseptically removed and enriched in de Man, Rogosa, Sharpe (MRS) broth (40 ml) (Hi-Media, India), homogenized and then inoculated at 37 °C for 24 h with constant homogenous shaking under aerobic conditions [26]. All the tubes showing turbidity were selected and further inoculated onto MRS agar (Hi-Media, India) plates and incubated for 24-72 h at 37 °C under aerobic conditions. Plates showing white and creamy colonies (presumptive for LAB) were selected, and individual colonies purified through three successive transfers on MRS medium.

The pure cultures were characterized as LAB by Gram staining, cell morphology, catalase, and coagulase reaction according to standard procedures [13]. Gram-positive, and catalase and coagulase-negative isolates were selected and stored at -20 °C in MRS broth plus 28% glycerol (El-Soda et al., 2003). The purified stocked cultures were resuscitated by sub-culturing twice in MRS broth before each use.

### Antagonistic activity

#### Agar well diffusion assay

The antagonistic effect of the LAB isolates against some pathogens was first determined by the agar well diffusion technique [62]. The LAB isolates were cultured in MRS broth at 37 °C overnight, and the targeted pathogens were also pre-cultured under the same conditions in Brain Heart Infusion (BHI) broth (Liofilchem, Italy). Exactly 200 μL of the test pathogens (10^7^ CFU/ml) was further spread onto the surface Mueller Hinton Agar (Biomark Lab, India) plates. Wells punctured into the inoculated plates were filled with 100 μL cell-free supernatant (CFS) obtained by centrifugation of LAB cultures at 6000 rpm for 10 min (Boeco, Germany). The plates were incubated at 37 °C for 24 h. Antagonistic activity of the LAB strains was assessed in terms of the formation of inhibition zones (mm) around the wells. This technique was conducted in triplicate for each LAB isolate and the mean result taken. The target pathogens tested were *Escherichia coli* ATCC 10536, *E. coli* O157: H7 ATCC 43894, *Enterococus faecalis* ATCC 51299, *Salmonella* Typhimurium ATCC 14028, *S.* Enteritidis ATCC 13098 and *Listeria monocytogenes* ATCC 19113.

#### Agar spot test

The antagonistic activity of the LAB strains was also conducted using the agar spot test as previously described by Armas et al. [27]. Overnight cultures of the target strains (pathogens) were diluted in BHI broth with 1 ml of each diluted culture (approximately 10^6^ CFU/ml) inoculated onto BHI agar plates. The excess culture was removed after 5 min of contact, and plates were left to dry for 30 min. MRS broth containing overnight cultures of the LAB strains to be tested for antagonistic activity were centrifuged (10 min at 15000 g), and 3 μl of CFSC of each LAB strain was spotted on the pathogen inoculated agar surface in triplicate. Plates were left for 5 min to absorb and then incubated aerobically at 37 °C for 24 h. Clear inhibition zone > 1 mm around a spot was measured and scored as positive.

### Safety of LAB probiotic strains

#### Haemolytic activity assay

The method of Maragkoudakis et al. [63] was used to determine the haemolytic activity of LAB isolates. Overnight cultures of LAB isolates grown in MRS broth were streaked onto blood agar base (Diagnostic Pasteur, France) plates containing 5% (v/v) of sheep blood and then incubated at 37 °C overnight. Haemolytic activities of the strains were recorded by the presence of Beta (β) haemolysis (indicated by a clear, colourless/lightened yellow zone surrounding the colonies depicting total lysis of RBC. Alpha (α) haemolysis (indicated by a small zone of greenish to brownish discoloration of the media, depicting reduction of haemoglobin to methemoglobin which diffuses around, and Gamma (ϒ) haemolysis (with no change observed in the media).

### Assessment of probiotic properties of LAB strains

#### Bile salt tolerance test

To assess the bile salt tolerance, overnight LAB cultures were resuspended in sterile PBS (pH 7.2) after centrifugation, and further adjusted to give 10^8^ CFU ml/L which was added into fresh MRS broth containing 0.3% bile salt (Merck KGaA, Germany) (w/v), and subsequently incubated for 6 h. The viability of cells was determined after 0, 3 and 6 h incubation by serial dilution and plating onto MRS agar [26].

### Simulated gastric juice survivability test

#### Preparation of simulated gastric juice

As previously described by Corcoran et al [64], simulated gastric juice was prepared with modifications. The formulation was devoid of proteose peptone because it may serve as free amino acid (L-glutamate,) source, which may consequently enhance bacterial growth by extruding protons from the cell.

#### Simulated gastric juice survivability test with and without lysozyme (pH 2)

For each LAB strain, 1 ml of fresh culture was resuspended in an equal volume of PBS as earlier explained. Pelleted cells were then resuspended in 5 ml volume of simulated gastric juice (with and without lysozyme) and then incubated at 37 °C for 90 min with constant stirring. At different time intervals of 0, 30, 60, and 90 min, samples were taken and serially diluted in maximum-recovery diluent up to 10^− 8^, and finally seeded on MRS agar plates, and incubated at 37 °C for 48 h [64].

#### Adhesion of LAB strains to chicken ileum epithelial cells

The LAB strains were tested for adherence to chicken epithelial cells as previously described [37] with modifications. The entire GIT was removed from chicken immediately after slaughter from a local abattoir and transported to the laboratory in the icebox. Gut contents were removed aseptically, and ileal segments were opened, repeatedly washed with PBS and held in PBS at 4^0^C for half an hour, to loosen the surface mucus. The washed ileum was divided into four small pieces (1cm^2^/1cm^2^), and each was incubated in cell suspension of LAB strains (10^9^ CFU/mL PSB) at 37°C for 90 min. At 0, 30, 60, and 90 min time intervals, samples were taken and screened for adherence. Non-adherent bacteria were removed by gently washing of incubated ileum with PSB, then macerated and finally, serially diluted in a maximum-recovery diluent, and subsequently plated onto MRS agar plates and incubated at 37^o^C for 24 hrs.

#### Phenol tolerance test

Phenol tolerance ability of LAB strains was determined by growing the strains in MRS broth containing increasing concentration (0.1–0.4%) of phenol [43]. After sterilization, each tube containing MRS broth with specific phenol concentration was inoculated with 1% (v/v) of fresh overnight cultures of LAB strains and incubated at 37^0^C for 24 h. Strains viability was assessed by measuring the absorbance by spectrophotometer (PG instruments, UK) at 620 nm after incubation. The experiment was repeated thrice.

#### Temperature and NaCl tolerance assay

Overnight LAB cultures (1% v/v) were inoculated into MRS broth and incubated at different temperatures of 4, 25, 37, 45 and 55 °C respectively for 24 h. Their growths were afterward determined by measuring their turbidity using the spectrophotometer at 600 nm, and subsequently seeded on MRS agar plates and incubated for 24 – 48 h at 37 °C. The appearance of LAB colonies on MRS agar plates corresponded and confirmed their growth in MRS broth [65]. Similarly, overnight LAB cultures (1% v/v) were inoculated into MRS broth containing increasing concentration of NaCl (0.5, 2.0, 4.0, 6.5 and 10.0%) and incubated overnight at 37 °C. Strains viability was assessed by measuring the absorbance at 600 nm. The experiment was carried out in triplicate.

#### Competitive adherence of LAB strains and pathogens to chicken ileum epithelial cells

Competitive pathogens exclusion is one of the primary mechanisms used by LAB in GIT. A piece of chicken ileum was prepared as previously described above and suspended in equal volumes of individual LAB strain and each pathogen (10^9^ CFU/mL PSB) and then incubated with at 37 °C for 90 min. Samples were taken and screened for competitive adherence 0, 30, 60, and 90 min intervals. Non-adherent bacteria from each piece of the incubated ileum was removed by gently washing with PSB, and then marcerated and subsequnetly, serially diluted in maximum-recovery diluent. Each diluent was plated onto MRS agar plates for LAB, MacConkey agar (HiMedia, India) plates for both *E. coli* and *E. coli* O157:H7 and *Salmonella Shigella* Agar (Liofilchem, Italy) plates for *S*. Typhimurium and *S*. Enteritidis respectively and incubated at 37 °C for 24 h for enumeration.

### Cell surface characteristics

#### Auto-aggregation assay

With some modifications, the method of Polak-Berecka et al. [66] was used in determining the auto-aggregation ability of LAB strains. LAB strains were pelleted in PBS (pH 7.2) (as previously described), and adjusted to get 10^8^ CFU ml/L in the same solution. Exactly 5 mL of bacterial suspension was vortexed (Vision Scientific, Korea) for 10 s, and the absorbance measured by the spectrophotometer at 600 nm (OD_i_), and then incubated for 2 h at 37 °C. The absorbance of the supernatant after 2 h of incubation was then measured (OD_2h_). The auto-aggregation coefficient (AC) was determined according to the formula below:

$$ A{C}_t\left(\%\right)=\left[1-\left(O{D}_{2h}/O{D}_i\right)\right]\ X\ 100 $$

$$ Given:O{D}_i= initial\ optical\ density\ of\ the\ microbial\ suspension\  at\ 600\; nm $$

$$ O{D}_{2h}= optical\ density\ of\ the\ microbial\ suspension\  at\ 600\; nm\  after\ 2\;h $$

#### Co-aggregation assay

Co-aggregation assay was conducted as previously described [27, 66]. The LAB isolates grown in MRS broth were harvested by centrifugation at 6000 x *g* for 15 min, washed twice and resuspended with sterile PBS (pH 7.2) and adjusted to 10^8^ CFU ml/L in the same solution. An equal volume, 2 ml of each isolate and each pathogen cultures were mixed, vortexed and incubated for 2 h at 37 °C. Each control tubes contained 4 mL of each bacterial suspension (i.e., the probiotic strain and the pathogen). The absorbance of each mixed suspension was then measured at 600 nm (ODmix) and compared to those of the control tubes containing the probiotic strain (ODstrain) and the specific pathogen (ODpathogen) at 2 h of incubation. co-aggregation was calculated using the formula below:

$$ Co- aggregation\ \left(\%\right)=\left[1- ODmix/\left( ODstrain+ ODpathogen\right)/2\right]x100 $$

#### LAB cell surface hydrophobicity assay

The cell surface hydrophobicity of LAB cells was assayed according to the method described previously by Abbasiliasi et al. [65]. Three tubes each containing 3 mL of each LAB strain cells suspension in PBS (pH 7.2) at 10^8^ CFU/mL were each mixed with n-hexadecane (1 mL) (a solvent) and then vortexed for 1 min. The mixture was subsequently allowed to separate into two phases by standing for 5–10 min. The OD (at 600 nm) of the aqueous phase was measured with a spectrophotometer. Bacterial affinity to solvent (n-hexadecane) (BATS) (hydrophobicity) was expressed using the equation below:

$$ BATS\ \left(\%\right)=\left(1-{A}_{10\mathit{\min}}/{A}_{0\mathit{\min}}\right)\ x\ 100 $$

Where, A_10min_ is the absorbance at t = 10 min, and A_0min_ is the absorbance at t = 0 min.

#### α–Glucosidase inhibitory activity of LAB strains

With slight modifications, the procedure of Kim et al. [50] was used to determine the inhibitory activity of α–glucosidase by LAB strains. Overnight culture of each LAB strain was centrifuged for 15 min at 4000×g and resuspended in PBS (50 μl). Exactly 3 mM *p-*nitrophenol-αD-glucopyranoside (*p*NPG, 50 μL) and the enzymatic reaction was allowed to proceed at 37°C for 30 min and finally stopped by the addition of 50 μL of 0.1 M Na_2_CO_3_, and the absorption released of Nitrophenol was measured at 405 nm using a microplate reader. The formula; (1-A/B) x 100 was used to calculate the inhibition of α-glucosidase activity of LAB strains, where A was the absorbance of the reactants with the sample, and B was the absorbance of the reactants without the sample (negative control). Acarbose was used as the standard reference (positive control).

#### Characterization of LAB antimicrobial substances

LAB strains with probiotic potentials were selected and further tested for the production antimicrobial substance, mainly bacteriocins, organic acids and hydrogen peroxides using the agar well diffusion technique as previously described [67] with modifications. Overnight cultures of LAB grown in MRS broth were centrifuged at 6000 g for 10 min, and the supernatants were collected and divided into four treatments: one was heat treated (boiled) for 10 min, the second was neutralized to pH 7 with 6 N NaOH (Fisher), the third was treated with 0.5 mg/ml catalase (Hi-media) and the fourth was untreated. These supernatants were subsequently filter sterilized (0.22 μm), and 100 μl was placed into wells bored in agar plates inoculated with 1% (v/v) overnight cultures of indicator pathogens as previously listed. The plates were further incubated at 37 °C overnight, and diameter of inhibition zones were measured (mm).

#### Antibiotic susceptibility test

The LAB isolates were examined for antimicrobial susceptibility, using the agar disc diffusion method [68]. The LAB strains to be tested were grown in fresh MRS broth at 37 °C overnight. The bacterial suspensions were matched to McFarland’s standard 2 (10^8^ CFU/mL) and subsequently spread onto the surface of the MRS agar plates using a sterile cotton swab. Commercially available antibiotic discs (Hi-Media, Mumbai) including penicillin G (2 units), ceftriaxone (30 μg), ampicillin (25 μg), vancomycin (30 μg), oxacillin (μg), streptomycin (10 μg), chloramphenicol (30 μg), gentamicin (10 μg), erythromycin (10 μg), tetracycline (10 μg), novobiocin (30 μg) and ciprofloxacin (10 μg) were aseptically placed on the surface of the dried inoculated agar plates, and then incubated for 24 h at 37 °C. Clear zones of microbial growth inhibition around each antibiotic were measured using a transparent ruler after 24 h incubation. Isolates were categorized as sensitive (≥21 mm), intermediate (16–20 mm), or resistant (≤ 15 mm) as previously assessed [65].

#### Biochemical identification of LAB strains using API 50 CHL

The carbohydrate fermentation profiles of most promising LAB probiotic strains were investigated using API 50 CH strips and API CHL medium according to the manufacturer’s instruction (API system, BioMèrieux, France). Overnight cultures of LAB grown in MRS broth were pelleted after washing twice with sterile PBS, and were re-suspended in API 50 CHL medium, using sterile pipettes. With subsequent mixing, the homogenized cells suspension were transferred into each of the 50 wells on the API 50 CH strips. The strips were covered as recommended and incubated at 30°C. Changes in color were monitored after 24 and 48 hrs of incubation. Results were represented by a positive sign (+) while a negative sign (−) was designated for no change. The apiweb™ Software version 5.0 (BioMèrieux, France) was used according to manufacturer’s instruction in the interpretation of the results.

#### Molecular identification by 16S rRNA sequencing

The molecular identification of LAB strains was conducted by 16S rRNA amplification, sequencing and analysis, using the universal forward and reverse primers 27F: AGAGTTTGATCMTGGCTCAG and 1492R: TACGGCTACCTTGTTACGACTT with 1500 bp product [29]. PCR reactions were conducted using a total volume of 20 μl, containing 10 μl of NZYTaq 2× Green Master Mix, 0.5 μl each of forward and reverse primers, 6 μl of DNase free water and 2 μl of DNA template. The amplification protocol of Shokryazdan et al. [29] was adopted for this study. After amplification, 10 μl of PCR products were analyzed for electrophoresis and then visualizaed by transillumination under UV light using ImageMaster (Pharmacia Biotech, UK). The PCR products with 1.5 kb as the expected size were purified and sequenced. The sequence data obtined were further compared with the database in the Genbank using the basic local alignment search tool (BLAST) fot the final idetification of the LAB strains.

### Statistical analysis

All measurements were repeated independently in triplicate, and results were expressed as mean ± standard deviation (SD). Data obtained were statistically analysed using GraphPad Prism version 5.0 for Windows (GraphPad Software, San Diego, CA, USA). One-way analysis of variance was used to study significant difference between means, with significance level at *P* < 0.05. Duncan’s multiple ranges or t-student test was used, when necessary, to discriminate differences between means. Differences were considered statistically significant at *p* < 0.05.

## Data Availability

All 16S sequences obtained in this study were deposited in NCBI with the accession numbers MK953805- MK953809.

## Abbreviations

16SrRNA: 16S ribosomal ribonucleic acid
CFS: Cell free supernatant
CFU/ml: Colony forming unit per millilitre
DNA: Deoxyribonucleic
GIT: Gastrointestinal tract
LAB: Lactic acid bacteria
MRA: Multiple antibiotic resistance
MRS: De Man, rogosa and sharpe
OD: Optical density
PCR: Polymerase chain reaction
spp: Species
